# Evaluation of Sperm Count Improvement Following Microsurgical Varicocelectomy

**DOI:** 10.7759/cureus.89427

**Published:** 2025-08-05

**Authors:** Mir Abid Jan, Syed Zia Ur Rahman, Khalil Ur Rehman

**Affiliations:** 1 Andro-Urology Unit, Institute of Kidney Diseases, Hayatabad Medical Complex, Peshawar, PAK; 2 Urology, Naseer Teaching Hospital, Peshawar, PAK

**Keywords:** male infertility, microsurgical varicocelectomy, semen analysis, sperm count, varicocele

## Abstract

Background: Varicocele is a common, correctable cause of male infertility, often associated with impaired spermatogenesis. Microsurgical subinguinal varicocelectomy is considered the gold standard for varicocele repair, with documented benefits on semen quality, particularly sperm count.

Objective: The objective of this study was to evaluate the effect of microsurgical varicocelectomy on sperm count in infertile men diagnosed with clinical varicocele.

Methodology: This prospective observational study was conducted at the Institute of Kidney Diseases, Hayatabad Medical Complex, Peshawar, Pakistan, over six months (October 1, 2024, to March 31, 2025). A total of 100 male patients aged between 20 to 45 years with clinical Grade I-III varicocele and abnormal semen parameters were enrolled. Patients underwent microsurgical subinguinal varicocelectomy after baseline semen analysis. A follow-up semen analysis was performed three months postoperatively. Pre- and postoperative sperm counts were compared using paired t-tests. Subgroup analysis was performed based on varicocele grade and laterality.

Results: The mean preoperative sperm count was 12.5 ± 6.8 million/mL, which significantly increased to 20.2 ± 8.1 million/mL postoperatively (p < 0.001). Patients with Grade III varicocele showed the highest improvement (22.6 ± 9.1 million/mL, p = 0.003). No significant difference was observed based on laterality (p = 0.087).

Conclusion: Microsurgical varicocelectomy significantly improves sperm count in men with clinical varicocele, particularly in higher-grade cases. It should be considered an effective first-line intervention in the management of varicocele-associated male infertility.

## Introduction

Male infertility is a significant global health concern, contributing to approximately 50% of all infertility cases among couples [1]. Among the various etiological factors, varicocele, defined as a pathological dilatation of the pampiniform venous plexus of the spermatic cord, is recognized as the most common correctable cause of male infertility [2]. It is found in about 15% of the general male population, up to 35% of men with primary infertility, and even more frequently in cases of secondary infertility [3].

The underlying pathophysiology of varicocele-induced infertility is complex and not fully elucidated. Multiple mechanisms have been proposed, including increased scrotal temperature, venous stasis leading to hypoxia, retrograde flow of renal and adrenal metabolites, oxidative stress, and hormonal dysregulation affecting the hypothalamic-pituitary-gonadal axis [4,5]. These factors contribute to impaired spermatogenesis and deterioration in semen quality, particularly in sperm count, motility, and morphology.

Surgical correction of varicocele, varicocelectomy, has undergone substantial refinement, with the microsurgical subinguinal approach now considered the gold standard. This technique offers favorable outcomes in terms of improved semen parameters, reduced complication rates, and lower recurrence rates [6,7]. While numerous studies have demonstrated the benefits of microsurgical varicocelectomy, outcome variability remains due to differences in patient selection, surgical approach, and follow-up protocols [8-11].

Given the high prevalence of varicocele among infertile men and the continuing debate regarding the extent of fertility restoration following surgery, further clarification is needed, especially in underrepresented populations. Regional data using standardized protocols remains sparse. Furthermore, sperm count remains a critical parameter in assessing male fertility potential, both for natural conception and success with assisted reproductive techniques.

The objective of this study was to evaluate the impact of microsurgical subinguinal varicocelectomy on semen quality in infertile men with clinically diagnosed varicocele. Specifically, the study aimed to assess changes in sperm count before and after surgery, examine whether varicocele grade or laterality influenced postoperative outcomes, and provide regional clinical data to support the role of microsurgical varicocelectomy as a first-line treatment option for varicocele-associated male infertility.

## Materials and methods

This was a prospective observational study conducted at the Department of Urology, Institute of Kidney Diseases, Hayatabad Medical Complex, Peshawar, Pakistan, from October 1, 2024, to March 31, 2025. Ethical approval was obtained from the Institutional Review Board (IRB) of Hayatabad Medical Complex, Medical Teaching Institution, Peshawar (Approval No: 1740, dated 02-09-2024).

The sample size was calculated using the OpenEpi online calculator, version 3.01 (Dean AG, Sullivan KM, Soe MM, 2013). OpenEpi: Open Source Epidemiologic Statistics for Public Health; http://www.OpenEpi.com) with a 95% confidence level and 80% power. Based on previous studies reporting post-varicocelectomy improvements in sperm count in approximately 60% to 70% of cases, the required sample size was estimated to be 100 patients [12]. A non-probability consecutive sampling technique was used to enroll eligible participants.

Inclusion criteria consisted of male patients aged 20-45 years with clinical varicocele (Grade II or III) [13], confirmed by physical examination and Doppler ultrasonography, and abnormal semen parameters (specifically low sperm count) documented in at least two semen analyses. Patients planning to undergo microsurgical varicocelectomy and willing to provide informed consent and comply with follow-up were included. Exclusion criteria comprised subclinical varicocele (detected only on Doppler), other known causes of infertility (e.g., obstructive azoospermia, genetic disorders, hormonal abnormalities), history of prior scrotal or varicocele surgery, active genitourinary infections or systemic illnesses affecting fertility, and recent use (within six months) of hormonal or antioxidant therapy.

Eligible participants were enrolled from the Department of Urology. A structured form captured demographic and clinical data, including age, BMI, varicocele grade, and laterality. Varicocele was graded clinically using the Dubin-Amelar classification [14]. Scrotal Doppler ultrasonography was used to confirm the diagnosis by assessing venous reflux and diameter, though not for grading. Subclinical cases were excluded to maintain diagnostic uniformity.

Sperm count (million/mL) was the primary outcome measure. Other semen parameters and hormonal profiles were not included due to resource and follow-up limitations but are acknowledged as important for future studies. Semen samples were collected via masturbation after two to seven days of abstinence and analyzed within 60 minutes per WHO 2021 guidelines [15]. Two baseline semen analyses were conducted at least two weeks apart.

All patients underwent microsurgical subinguinal varicocelectomy performed by an experienced consultant urologist. Postoperative care followed standard protocols. Follow-up semen analysis was done at three months using the same WHO criteria and by the same laboratory personnel to reduce inter-observer variability. No control group was included, as the primary aim was to assess within-subject changes following standardized intervention in a clinically indicated setting.

Data were analyzed using IBM SPSS version 21 (IBM Corp., Armonk, NY). Continuous variables (e.g., age, sperm count) were reported as mean ± SD; categorical variables (e.g., varicocele grade, laterality) as frequencies and percentages. The Shapiro-Wilk test assessed normality; Levene’s test checked variance homogeneity. Paired t-tests compared pre- and post-operative sperm counts. Subgroup analyses by varicocele grade used one-way ANOVA with post-hoc testing; laterality comparisons used independent t-tests. If normality assumptions were not met, non-parametric equivalents were applied (Wilcoxon signed-rank, Kruskal-Wallis, and Mann-Whitney U tests). A p-value < 0.05 was considered statistically significant.

Written informed consent was obtained from all participants after explaining the study objectives and procedures. Confidentiality was ensured by assigning anonymized codes and securing data in password-protected files accessible only to the research team.

## Results

A total of 100 patients were included in the study. The mean age of participants was 29.6 ± 5.4 years, and the average BMI was 24.2 ± 3.1 kg/m². Regarding varicocele severity, 40% of patients had Grade III varicocele, 38% had Grade II, and 22% had Grade I. In terms of laterality, 82% of patients presented with left-sided varicocele, while 18% had bilateral involvement. The mean preoperative sperm count was 12.5 ± 6.8 million/mL (Table 1).

**Table 1:** Demographic and baseline characteristics of the participants (n = 100)

Variable	Mean ± SD/Frequency (%)
Age (years)	29.6 ± 5.4
BMI (kg/m²)	24.2 ± 3.1
Varicocele grade	
Grade I	22 (22%)
Grade II	38 (38%)
Grade III	40 (40%)
Laterality	
Left-sided	82 (82%)
Bilateral	18 (18%)
Preoperative sperm count (million/mL)	12.5 ± 6.8

Following microsurgical varicocelectomy, there was a statistically significant improvement in sperm count. The postoperative sperm count increased to 20.2 ± 8.1 million/mL, compared to the preoperative value of 12.5 ± 6.8 million/mL. The mean difference was 7.7 ± 4.5 million/mL, and the result was highly significant (p < 0.001, paired t-test) (Table 2).

**Table 2:** Comparison of pre- and postoperative sperm count (n = 100) A paired t-test was used, and p≤ 0.05 is considered significant.

Measurement	Preoperative mean ± SD	Postoperative mean ± SD	Mean difference ± SD	t-statistic	p-value
Sperm count (million/mL)	12.5 ± 6.8	20.2 ± 8.1	7.7 ± 4.5	17.11	<0.001

Subgroup analysis revealed that postoperative sperm count varied significantly with varicocele grade. Patients with Grade III varicocele showed the highest mean sperm count postoperatively (22.6 ± 9.1 million/mL), followed by Grade II (19.8 ± 6.7 million/mL) and Grade I (17.4 ± 5.2 million/mL), with a statistically significant difference (p = 0.003, one-way ANOVA). However, no statistically significant difference was found in postoperative sperm count between patients with left-sided (20.4 ± 7.9 million/mL) and bilateral varicocele (19.1 ± 8.5 million/mL) (p = 0.087, independent t-test) (Table 3).

**Table 3:** Postoperative sperm count by varicocele grade and laterality (subgroup analysis) For varicocele grade, a one-way ANOVA test was applied, and for laterality, an independent t-test was applied. p≤ 0.05 is considered significant.

Grouping variable	Postoperative sperm count (Mean ± SD)	Test statistic	p-value
Varicocele grade			
Grade I	17.4 ± 5.2	F(2,97)=5.98	0.003
Grade II	19.8 ± 6.7		
Grade III	22.6 ± 9.1		
Laterality			
Left-sided	20.4 ± 7.9	t(98)=1.74	0.087
Bilateral	19.1 ± 8.5		

## Discussion

This study aimed to evaluate the effect of microsurgical varicocelectomy on sperm count among infertile males with clinically diagnosed varicocele. The findings demonstrate a statistically significant improvement in sperm count following microsurgical intervention, with a mean increase of 7.7 ± 4.5 million/mL postoperatively (p < 0.001). These results are consistent with a large body of evidence supporting the effectiveness of microsurgical varicocelectomy in improving semen parameters [8, 16, 17].

Importantly, impaired semen quality is a multifaceted condition encompassing abnormalities in sperm concentration (oligospermia), motility (asthenozoospermia), morphology (teratozoospermia), and combined defects (oligoasthenoteratozoospermia (OAT syndrome)). Although our study focused on sperm count as the primary outcome, previous studies have shown that microsurgical varicocelectomy can lead to improvements across multiple semen parameters. For instance, Fu et al. (2024) found that the procedure significantly enhanced sperm concentration, motility, and morphology, particularly in men with clinically palpable varicocele and multiple semen abnormalities [18].

Panah et al. (2024) further demonstrated that patients with more severely impaired semen profiles, particularly those with moderate to severe oligospermia, experienced greater improvements in postoperative semen quality compared to those with only borderline or mild abnormalities [19]. This observation aligns with our findings, where participants with markedly low baseline sperm counts showed a robust mean gain post-surgery, suggesting that the degree of preoperative impairment may predict the magnitude of benefit.

Similarly, Kaltsas et al. (2022) reported a mean post-varicocelectomy sperm count increase of 15 million/mL and emphasized that men with OAT syndrome particularly benefit from the subinguinal microsurgical approach [20]. Wang et al. (2021) also supported this by noting that this technique leads to superior improvements in overall semen quality, likely due to better preservation of surrounding structures [8].

In our study, subgroup analysis revealed that patients with higher-grade varicocele (Grade III) experienced more pronounced improvements in sperm count (22.6 ± 9.1 million/mL) compared to those with lower grades, further supporting the idea that the extent of clinical disease correlates with therapeutic gain. On the other hand, laterality (left-sided vs. bilateral) did not show a statistically significant difference in outcomes (p = 0.087), though left-sided varicoceles were more prevalent, consistent with known anatomical patterns [21, 22].

While some studies, such as Lamb et al. (2023), have raised concerns about the correlation between improved semen parameters and actual fertility outcomes [23], it is well-established that enhanced sperm quality, especially in terms of count, motility, and morphology, plays a vital role in both natural conception and success with assisted reproductive techniques (ART). Therefore, the improvements observed in our study are clinically meaningful.

The use of microsurgical subinguinal varicocelectomy as a frontline treatment option for infertile men with clinical varicocele, particularly those with significant semen abnormalities. By improving key semen parameters, this approach may reduce the need for invasive or costly ART, thereby lessening the emotional and financial burden on affected couples.

Despite its strengths, this study has several limitations. First, it was a single-center study with a relatively modest sample size (n = 100), which may limit the generalizability of the findings to broader populations. Second, although sperm count was selected as the primary outcome due to its clinical relevance and consistency in laboratory reporting, other important semen parameters, such as motility, morphology, vitality, and hormonal profiles (e.g., follicle-stimulating hormone (FSH), luteinizing hormone (LH), and testosterone), were not evaluated and should be incorporated in future studies to provide a more comprehensive assessment of fertility potential. Third, pregnancy and live birth outcomes were not assessed, preventing a direct correlation between improved semen parameters and actual fertility success. Additionally, the follow-up period was limited to three months, and longer-term outcomes remain unknown. Lastly, the absence of a control group or comparison with alternative treatment modalities (e.g., laparoscopic or embolization techniques) limits the ability to exclude confounding variables or contextualize the effectiveness of the microsurgical approach. Future research should include larger, multicenter cohorts with extended follow-up and broader outcome measures, including reproductive outcomes, to validate and extend the current findings.

## Conclusions

This study demonstrated that microsurgical subinguinal varicocelectomy significantly improves sperm count in infertile men with clinical varicocele, with the greatest benefit observed in those with higher-grade varicoceles. The procedure offers a valuable, minimally invasive treatment option that can enhance semen quality and potentially improve fertility outcomes. While the results are encouraging, further large-scale, multicenter studies with longer follow-up and inclusion of additional fertility parameters such as motility, morphology, and pregnancy rates are needed to confirm the long-term efficacy and reproductive benefits of this surgical intervention.
